# Pap test recency and HPV vaccination among Brazilian immigrant women in the United States: a cross-sectional study

**DOI:** 10.1186/s12889-024-19424-7

**Published:** 2024-07-22

**Authors:** Mary L. Greaney, Steven A. Cohen, Jennifer D. Allen

**Affiliations:** 1 Department of Public Health, University of Rhode Island, 25 West Independence Way, Kingston, RI 02181 USA; 2 Department of Community Health, Tufts University, 574 Boston Avenue, Medford, MA 02155 USA

**Keywords:** Immigrant, Pap test, HPV, Vaccination, Brazilian, Health behaviors

## Abstract

**Background:**

The United States (U.S.) has a growing population of Brazilian immigrant women. However, limited research has explored Pap tests and human papillomavirus (HPV) vaccination among this population.

**Methods:**

Participants completed an online survey between July—August 2020. Bivariate analyses examined associations between healthcare-related variables (e.g., insurance, having a primary care provider) and demographics (e.g., age, education, income, marital status, years living in the U.S., primary language spoken at home) with 1) Pap test recency (within the past 3 years) and 2) HPV vaccination (0 doses vs. 1 + doses). Variables significant at *p* < 0.10 in bivariate analyses were included in multivariable logistic regression models examining Pap test recency and HPV vaccination.

**Results:**

The study found that 83.7% of the sample had a Pap test in the past three years. Women who did not know their household income were less likely to be than women who reported a household income of < $25,000 (adjusted OR [aOR] = 0.34, 95% CI: 0.12, 0.95). Women who had seen a healthcare provider in the past year were more likely to have had a Pap test within the last three years than those who had not seen a provider in the past year ([aOR] = 2.43, 95% CI: 1.32, 4.47). Regarding HPV vaccination, 30.3% of respondents reported receiving one or more doses of the HPV vaccine. The multivariable logic regression models determined that women aged 27 -45 (aOR = 0.35, 95% CI: 0.18, 0.67) were less likely than women aged 18–26 to have been vaccinated against HPV). and that women with a PCP were more likely to be vaccinated than those without a PCP (aOR = 2.47. 95% CI:1.30, 4.59).

**Conclusion:**

This study found that Brazilian immigrant women in the youngest age groups (21 – 29) for Pap test, 18- 26 for HPV vaccination) had somewhat better rates of Pap screening and HPV vaccination than the general U.S. population. This study adds new information about cervical cancer prevention and control behaviors among Brazilian immigrant women.

## Background

There is a growing Brazilian population in the United States (U.S.), and the U.S. is now home to the largest Brazilian population outside of Brazil. The foreign-born Brazilian immigrant population in the U.S. increased from 340,000 to 502,000 between 2010 and 2019 [32]. This number likely is an underestimate as it is estimated that approximately 70% of Brazilian immigrants in the U.S. are undocumented [19], which is viewed as an important social determinant of health. In general, immigrants are less likely to have health insurance, access to health care, and perceive discrimination when seeking healthcare services [24]. Although Brazilians are generally classified as Hispanic in U.S.-based studies, Brazilians in the U.S. often do not identify as Hispanic due to speaking Portuguese and having different cultural origins [25]. They may have needs that differ from other Hispanic groups that are not well understood and, as a result, not addressed.

It is estimated that there will be 13,820 new cases of invasive cervical cancer and 4,360 cervical cancer deaths in the U.S. in 2024 [4]. In Brazil, cervical cancer is the third most common cancer and the fourth leading cancer death in women. Corrêa, et al., [11], and it is estimated prevalence of HPV infection is 25.4% [10]. In the U.S., it is recommended that women aged 21 to 29 years of age be screened for cervical cancer every three years via a Pap smear test and that women 30 to 65 years be screened every three years with a Pap test and every five years with high-risk HPV testing, or every five years with both [12].

The HPV vaccine became available in the U.S. in 2006. Recommendations regarding vaccination in the U.S. were initially for girls starting at 11 or 12 years, with the expansion of guidelines to include vaccination starting at age 9, and a “catch-up” vaccination for females between 13 and 26 years of age who have not yet been fully vaccinated [6, 23]. HPV vaccination was recently approved in the U.S. for adults ages 27 to 45 that have not been vaccinated. However, HPV vaccination for this age group is not routinely recommended. Rather, it is recommended that clinicians discuss HPV vaccination with those most likely to benefit, using a shared decision-making approach, which entails a discussion of individual risks, benefits, and limitations of the HPV vaccine in this age group and patient preferences [21]. Historically, the HPV vaccine has been administered as a series of two or three doses, depending on the age of initiation [21]. The HPV vaccine first became available in Brazil in 2014 for girls ages 9–13 years [5]. Both Pap tests and HPV vaccination are available free of charge in Brazil [11]. The World Health Organization recently issued guidance that one dose may offer “solid” protection (comparable to two doses) among 9 -20-year-olds [34]. However, two doses are recommended for women over 21 [34]. Data for this study were collected before the recent WHO guidance.

HPV vaccination rates in the U.S. are subpar, with notable disparities [16, 20]. For example, in 2018, 42.1% of non-Hispanic White women aged 18–26 had received one or more doses of the HPV vaccine compared to 36.1% of Hispanic women in this age group [6]. There is limited research examining HPV vaccination among immigrant adults in the U.S. An analysis of data from the 2014 and 2015 National Health Interview Survey determined that among men and women 18–26 years old, the initiation of HPV vaccination (≥ 1 dose) was nearly double among the U.S.-born survey respondents (28.6%) compared to foreign-born respondents (14.5%) [7]. There is minimal research examining HPV vaccination among Brazilian Immigrants in the U.S. The only study we identified involving Brazilian immigrants focused on parents and found that most respondents (93.6%) were aware of HPV, yet only 74.5% were aware of the HPV vaccine [18]. Given the growing Brazilian immigrant population in the U.S., and the importance of Pap testing and HPV vaccination in reducing morbidity and mortality due to cervical cancer, the primary aim of this cross-sectional study was to describe the prevalence of having a PAP test within the past three years and HPV vaccination. A secondary aim was identifying demographic factors associated with these primary and secondary preventive measures.

## Methods

### Study procedures

We have described the study methods elsewhere [3]. In brief, we used Brazilian social media (e.g., Facebook, WhatsApp) and collaborated with community social service providers and advocacy organizations in Massachusetts to recruit participants for this cross-sectional study. Eligibility requirements included being female,18 years or older, being born in Brazil, and currently residing in the U.S. Individuals interested in participating were directed to the study URL, where they accessed the informed consent documents in English or Portuguese. Before completing the online survey, participants were required to indicate that they had reviewed informed consent information and agreed to study participation. The survey was conducted between July and August 2020. Participants received a $20 Amazon gift card after completing the survey. Tufts University Institutional Review Board approved this study (protocol #0001838).

### Measures

#### Cervical cancer screening

As part of the online survey, respondents reported if they had ever had a Pap test (yes, no, don’t know/not sure) and how long it had been since their last test (never, within the past year, within the past 2 years, within the past 3 years, within the past 4 years, within the past 5 years, 5 or more years ago). We used these two variables to determine if respondents had a Pap test within the last three years (yes, no). Survey items were from the Behavioral Risk Factor Surveillance System (BRFSS) [8].

#### HPV vaccination

Respondents also reported if they had received the HPV vaccine (yes, no, don’t know/not sure). Respondents who reported receiving the vaccine were asked how many doses they had (1, 2, 3, don’t know/not sure). We created a dichotomous HPV vaccination variable for analysis (0 doses vs. 1 + doses), with 1 + doses being conceptualized as “being vaccinated.”

#### Healthcare-related variables

Participants reported if they had health insurance (yes, no, not sure) and type of insurance (plan purchased through an employer or union, plan that [you] or another family, Medicare, Medicaid, or other state program (MassHealth, Medi-Cal), TRICARE (Formerly CHAMPUS), Veterans’ Administration [V.A.] or Military). We combined these two items to create a single variable measuring health insurance coverage (none, public, private, don’t know/missing). Participants also reported if they had a primary care provider that they identified as their own (yes, no) and the last time they had seen a healthcare provider (within the past year, within the past 2 years, within the past 3 years, within the past 4 years, within the past 5 years, 5 or more years ago). We dichotomized this variable to have seen a healthcare provider within the last year (yes, no).

#### Demographic characteristics

Respondents reported their race/ethnicity (White, Black, Asian, Indigenous, Multiracial Other, Pardo [mixed]). We combined multiracial, Indigenous, Multiracial, and other races into one group due to the small cell sizes. Respondents also reported their education level (incomplete primary education, complete primary education, incomplete secondary education, complete secondary education, incomplete tertiary education, complete tertiary education, don’t know) using items from the Brazilian Census [17]. Due to small cell sizes, we created three education groups for analysis: incomplete secondary education or less, complete secondary and incomplete tertiary education, and complete tertiary education.

Respondents also reported their age (continuous), and this information was used to create age groups. Participants also reported their household income (less than $25,000, $25,001—$50,000, $50,0001- $75,000, < $100,000, don’t know). Lastly, using items from the BRFSS [8], participants reported if they identified as Latina (yes, no), language spoken at home (English only, some English and some Portuguese, Portuguese only, other), and their marital status (married, living as married, single, divorced, windowed). For analysis, marital status was dichotomized as married/living (as married vs single/divorced/ widowed). Additionally, respondents reported the number of years they had lived in the U.S. Years lived in the U.S. to date was used as a continuous and categorical variable (< 1, ≥ 1–5, and 5 + years) in the analysis.

### Analysis

Two analytic samples were created: one to examine the recency of cervical cancer screening and a second to examine HPV vaccination. For the analysis examining Pap test recency, respondents between 18 and 20 and 65 + years of age were excluded from the analytic sample based on screening recommendations [12]. We then created the following age groups: 1) 21 – 29 years, 2) 30 – 39 years, 3) 40 – 49 years, and 50 – 64 years.

For the analysis examining HPV vaccination, we limited the analytic sample to respondents between 18–45 years of age, as the HPV vaccine was introduced in the US in 2006 for females between 9–26 years of age and in Brazil in 2014 (initially for girls 9–11). We then created two age groups (18—26 and 27—45) to align with The Advisory Committee on Immunization Practices recommendations [23]. Study participants aged 27–45 would possibly be eligible for HPV vaccination when younger, depending on whether they were living in the U.S, or Brazil.

We first calculated descriptive statistics for all variables, means and standard deviations for the continuous variables, and frequencies for categorical variables for both samples. We then conducted bivariate analyses to examine the associations between having had a Pap test within the last three years and healthcare-related variables (health insurance coverage and type, having a primary care provider, and having seen a healthcare provider in the last year) and the demographic measures (age, age group, education, household income, identifying as Latina, marital status, number of years living in the US (continuous and categorical), and primary language spoken at home) using chi-square tests, Fisher’s exact tests, independent sample t-tests, or Wilcoxon rank sum tests, as appropriate. Next, we included all variables significant at *p* < 0.10 in the bivariate analyses in a series of multivariable logistic regression models constructed to calculate the adjusted odds ratios (ORs) and 95% confidence intervals (CI) to assess associations between healthcare-related and demographic variables with being current with cervical cancer screening. Similar analyses examined HPV vaccination (0 doses vs.1 + doses). We conducted all analyses using SAS version 9.4 (SAS Institute Inc., Cary, North Carolina) statistical software and IBM SPSS version 29 (Armonk, NY).

## Results

### Sample characteristics

#### Pap recency sample

The sample (*n* = 338) had a mean age of 39.6 years (SD = 10.4 years, median 39, IQR [30–47]), and 18.6% were between 21 and 29 years of age. In total, 58.6% of respondents identified their race as White, while 77.8% identified as Latina. Nearly half (45.5%) spoke only Portuguese at home, and 36.4% had lived in the U.S. for five years or fewer (median 9 years, interquartile range (IQR) [4–19 years]). See Table 1 for additional demographic information about the sample.

**Table 1:** Demographic characteristics of the sample, overall and by Pap test within past three years

			**Pap test within past three years**				
**Characteristic**	**Overall** **(*n* = 338)**		**Yes** **(*n* = 283, 83.7%)**		**No** (*n* = 55, 16.3%)		*P*-value**
**Age (mean, SD in years)**	39.7	10.4	39.5	10.1	40.8	10.7	0.40
	n	%^a^	n	%^a^	n	%^a^	
**Age groups**							
21–29	63	18.6	55	19.4	8	14.5	0.85
30–39	110	32.5	92	32.5	18	32.7	
40–49	98	29.0	81	28.6	17	30.9	
50–64	67	19.8	55	19.4	12	21.8	
**Race**							
Black	23	6.8	20	7.1	3	5.5	0.82
Pardo	78	23.1	63	22.3	15	27.3	
White	198	58.6	168	59.4	30	54.5	
Multiracial, indigenous, or another race	39	11.5	32	11.3	7	12.7	
**Identify as Latina**							
Yes	263	77.8	217	76.7	46	83.6	0.46
No	48	8.0	43	15.2	4	9.1	
Don’t know	27	14.2	23	8.1	5	7.3	
**Years lived in the U.S. (mean, SD in years)**	11.9	9.1	11.9	9.0	12.0	9.2	0.93
**Years lived in the U.S. (Categorical)**							
< 1	24	7.1	20	7.1	4	7.5	0.71
> 1–5	99	29.5	81	28.6	18	34.0	
> 5	213	63.4	182	64.3	31	58.5	
**Marital status**							
Married/living as married	248	73.4	211	74.6	37	67.3	0.26
Single/divorced/widowed	90	26.6	72	25.4	18	32.7	
**Household Income**							
< $25,000	107	31.7	90	31.8	17	30.9	0.05
$25,001–50,000	63	18.6	51	18.0	12	21.8	
$50,001–75,000	52	15.4	44	15.5	9	14.5	
$75,001–100,000	43	12.7	36	12.7	7	12.7	
> $100,000	51	15.1	48	17.0	3	5.5	
Don’t know/missing	22	6.5	14	4.9	8	14.5	
**Education**							
Incomplete secondary education or less	57	17.1	44	15.8	13	23.9	0.39
Complete secondary and incomplete tertiary education	113	33.6	97	34.9	16	29.4	
Complete tertiary education	165	49.3	140	50.3	25	45.5	
**Primary language spoken at home**							
English only	29	8.7	24	8.6	5	9.1	0.99
Portuguese only	152	45.5	127	45.5	25	45.5	
Some English/some Portuguese	153	45.8	128	45.9	25	45.5	
**Health insurance coverage and type**							
Public	119	35.2	98	34.6	21	23.6	0.65
Private	137	40.5	118	41.7	19	38,2	
None	65	19.2	52	18.4	13	34.5	
Don’t know/missing	17	5.0	15	5.3	2	3.5	
**Primary care provider**							
Yes	214	63.3	186	65.7	28	50.9	0.11
No	120	35.5	94	33.2	26	47.3	
Don’t know	4	1.2	3	1.1	1	1.8	
**Saw healthcare provider within past year**							
Yes	217	64.2	193	68.2	24	43.6	< 0.01
No	121	35.8	90	31.8	31	56.4	
**HPV Vaccine**							
Yes	69	25.0	61	26.8	8	16.7	0.14
No	207	75.0	167	73.2	40	83.3	

^a^Percent totals may not equal 100 due to missing data

**From chi square (or t-test for age and years in US)

#### HPV vaccination sample

The sample (*n* = 211) had a mean age of 33.7 years (SD = 7.5, median 35, IQR [28–40]), and 57.3% of respondents identified their race as White, while 75.4% identified as Latina. Nearly half (44.2%) spoke only Portuguese at home, and 45.9% had lived in the U.S. for five years or fewer (median 7, IQR [3–15 years]. See Table 2 for additional demographic information about the sample.

**Table 2:** Demographic characteristics of the sample, overall, and by HPV vaccination status (one or more doses vs. none)

**Characteristic**	**Overall** (*n* = 211)		**Vaccinated** (1 + doses) (*n* = 64, 30.3%)		**Not Vaccinated** (0 doses) (*n* = 147, 69.7%)		*P***-value**
**Age (mean, SD in years)**	33.7	7.5	30.9	7.8	34.9	7.1	< 0.01
	n	%*	n	%*	n	%*	
**Age groups**							
18–26	47	22.3	23	35.9	24	16.3	< 0.01
27–45	164	77.7	41	64.1	123	83.7	
**Race**							
Black	20	9.5	6	9.4	14	9.5	0.88
Pardo	49	23.2	14	21.9	35	23.8	
White	121	57.3	36	56.3	85	57.8	
Multiracial, indigenous, or another race	21	10.0	8	12.5	13	8.8	
**Identify as Latina**							
Yes	159	75.4	52	81.3	107	72.8	0.21
No	34	16.1	6	9.4	28	19.0	
Don’t know	18	8.5	6	9.4	12	8.2	
**Years lived in the U.S. (mean, SD in years)**	9.0	7.1	9.9	7.7	8.7	97.0	0.15
**Years lived in the U.S. categories**							
< 1	18	8.6	4	6.3	14	9.6	0.60
> 1–5	78	37.3	22	34.9	56	38.4	
> 5	113	54.1	37	58.7	76	52.1	
**Marital status**							
Married/living as married	139	65.9	30	46.9	109	74.1	< 0.01
Single/divorced/widowed	72	34.1	34	53.1	38	25.9	
**Household Income**							
< $25,000	74	35.1	23	35.9	51	34.7	0.34
$25,001–50,000	42	19.9	12	18.8	30	20.4	
$50,001–75,000	34	16.1	14	21.9	20	13.6	
$75,001–100,000	20	9.5	4	6.3	16	10.9	
> $100,000	30	14.2	6	9.4	24	16.3	
Don’t know/missing	11	5.2	5	7.8	6	4.1	
**Education**							
Incomplete secondary education or less	36	17.1	12	18.8	24	16.3	0.24
Complete secondary and incomplete tertiary education	71	33.6	26	40.6	45	30.6	
Complete tertiary education	104	49.3	26	40.6	78	53.1	
**Primary language spoken at home**							
English only	18	8.7	6	9.5	12	8.3	0.87
Portuguese only	92	44.2	29	46.0	83	43.4	
Some English/some Portuguese	98	47.1	28	44.4	70	48.3	
**Health insurance coverage and type**							
Public	71	33.6	24	37.5	47	27.9	0.15
Private	77	36.5	24	37.5	53	32.0	
None	51	24.2	10	15.6	41	36.1	
Don’t know/missing	12	5.7	6	9.4	6	4.1	
**Primary care provider**							
Yes	113	53.6	44	68.8	69	46.9	0.01
No	95	45.0	20	31.2	75	51.0	
Don’t know	3	1.4	0	0.0	3	2.0	
**Saw healthcare provider within past year**							
Yes	121	57.3	40	62.5	81	55.1	0.32
No	90	42.7	24	37.5	66	44.9	
**Pap test within the past 3 years**							
Yes	164	79.2	49	77.8	115	79.9	0.73
No	43	20.8	14	22.2	29	20.1	

### Pap test recency

In total, 83.7% of respondents had a Pap test in the past 3 years. As seen in Table 3, only income and having seen a healthcare provider within the past year were associated with Pap test recency in the bivariate analysis (all *p* values < 0.10). The multivariable logistic regression models determined that women who did not know their household income were less likely to have had a Pap test within the past three years than women who reported a household income of < $25,000 (adjusted odds ratio [aOR] = 0.34, 95% CI: 0.12, 0.95). Women who had seen a healthcare provider in the last year were more likely to have had a Pap test within the past three compared to those who had not seen a provider (aOR = 2.43, 95% CI: 1.32, 4.47).

**Table 3:** Odds ratios (OR) and 95% confidence intervals (CI) of having had a Pap test within the past three years

	Pap test within the last three years			
	Bivariate Model	*P*-value	Multivariable Logistic Regression Model	*P*-value
**Characteristic**	OR (95% CI)		OR (95% CI)	
**Age (per 1-year increase)**	0.99 (0.96, 1.02)	0.40		
**Age groups**				
21–29	Reference			
30–39	0.74 (0.30, 1.82)	0.53		
40–49	0.69 (0.28, 1.72)	0.43		
50–64	0.67 (0.25, 1.76)	0.41		
**Race**				
White	Reference			
Black	1.19 (0.33, 4.26)	0.79		
Pardo	0.75 (0.38, 1.49)	0.41		
Multiracial, indigenous, or another race	0.82 (0.33, 2.02)	0.66		
**Identify as Latina**				
Yes	0.67 (0.16, 2.74)	0.58		
No	Reference			
Don’t know	0.55 (0.21, 1.48)	0.23		
**Years lived in the U.S. per 1-year increase**	1.00 (0.97, 1.03)	0.93		
**Years lived in the U.S**				
< 1	Reference			
1- ≤ 5	0.90 (0.27, 2.96)	0.86		
5 +	1.17 (0.38, 3.67)	0.78		
**Marital status**				
Married/living as married	1.43 (0.76, 2.66)	0.27		
Single/divorced/widowed	Reference			
**Household Income**				
< $25,000	Reference			
$25,001- 50,000	0.80 (0.36, 1.81)	0.60	0.82 (0.35, 1.92)	0.65
$50,001- 75,000	1.04 (0.42, 2.59)	0.94	1.16 (0.44, 3.04)	0.76
$75,001–100,000	0.97 (0.37, 2.54)	0.95	0.96 (0.36, 2.57)	0.94
> $100,000	3.02 (0.84, 10.83)	0.09	2.47 (0.68, 8.97)	0.17
Don’t know	**0.33 (0.12, 0.91)**	**0.03**	**0.34 (0.12, 0.95)**	**0.04**
**Education**				
Incomplete secondary education or less	Reference			
Complete secondary and incomplete tertiary education	1.79 (0.79, 4.04)	0.160		
Complete tertiary education	1.66 (0.78, 3.51)	0.189		
**Primary language spoken at home**				
English only	Reference			
Portuguese only	1.06 (0.37, 3.04)	0.92		
Some English/some Portuguese	1.07 (0.37, 3.06)	0.91		
**Health insurance coverage and type**				
None	Reference			
Public	1.17 (0.54, 2.52)	0.69		
Private	1.55 (0.71, 3.38)	0.27		
Don’t know/missing	1.88 (0.38, 9.25)	0.44		
**Primary care provider**				
Yes	1.21 (0.12, 12.07)	0.87		
No	Reference			
** Saw healthcare provider within past year**				
Yes	**2.77 (1.54, 4.99)**	** < 0.01**	**2.43 (1.32, 4.47)**	**< 0.01**
No	Reference		Reference	

Bold font indicates *p* < 0.05

### HPV vaccination

In total, 30.3% of respondents reported having received one or more doses of the HPV vaccine. As seen in Table 4, age (continuous and categorical), marital status, and having a primary care provider were the only variables associated with having one or more doses of the HPV vaccine in the bivariate analysis (all p-values < 0.10). The multivariable logic regression models determined that women aged 27 -45 were less likely than women aged 18–26 to have been vaccinated against HPV (aOR = 0.35, 95% CI: 0.18, 0.67) and that women who reported having a primary care provider were more likely to be vaccinated than those without a primary care provider (aOR = 2.47. 95% CI:1.30, 4.59).

**Table 4:** Odds ratios (OR) and 95% confidence intervals (CI) of receiving the HPV vaccine

	HPV vaccination (1 + doses vs. none)			
	Bivariate Model	*P*-value	Multivariable Logistic Regression Model	*P*-value
**Characteristic**	OR (95% CI)		OR (95% CI)	
**Age (per 1-year increase)**	**0.93 (0.90, 0.97)**	** < 0.01**		
**Age groups**				
18–26	Reference		Reference	
27–45	**0.35 (0.18, 0.68)**	** < 0.01**	**0.40 (0.19, 0.84)**	**0.02**
**Race**				
White	Reference			
Black	1.01 (0.36, 2.84)	0.98		
Pardo	0.94 (0.45, 1.96)	0.88		
Multiracial, indigenous, or another race	1.45 (0.56, 3.81)	0.45		
**Identify as Latina**				
Yes	0.44 (0.17, 1.13)	0.09		
No	Reference			
Don’t know	1.03 (0.37, 2.90)	0.96		
**Years lived in the U.S. per 1-year increase**	1.02 (0.98, 1.06)	0.30		
**Years lived in the U.S**				
< 1	Reference			
1- ≤ 5	1.38 (0.41, 4.64)	0.61		
5 +	1.70 (0.52, 5.54)	0.38		
**Marital status**				
Married/living as married	**0.31 (0.17, 0.57)**	** < 0.01**	**0.35 (0.18, 0.67)**	**< 0.01**
Single/divorced/widowed	Reference		Reference	
**Household Income**				
< $25,000	Reference			
$25,001- 50,000	0.89 (0.39, 2.04)	0.78		
$50,001- 75,000	1.55 (0.67, 3.60)	0.31		
$75,001–100,000	0.55 (0.17, 1.84)	0.34		
> $100,000	0.55 (0.20, 1.54)	0.26		
Don’t know	1.85 (0.51, 6.68)	0.35		
**Education**				
Incomplete secondary education or less	Reference			
Complete secondary and incomplete tertiary education	1.16 (0.50, 2.69)	0.74		
Complete tertiary education	0.67 (0.29, 1.52)	0.33		
**Primary language spoken at home**				
English only	Reference			
Portuguese only	0.92 (0.31, 2.70)	0.88		
Some English/some Portuguese	0.80 (0.27, 2.34)	0.68		
**Health insurance coverage and type**				
None	Reference			
Public	2.09 (0.90, 4.89)	0.09		
Private	1.86 (0.80, 4.31)	0.15		
Don’t know/missing	**4.10 (1.09, 15.44)**	**0.04**		
**Primary care provider**				
Yes	**1.93 (1.09, 3.43)**	**0.03**	**2.47 (1.30, 4.59)**	**0.01**
No	Reference		Reference	
**Saw healthcare provider within past year**				
Yes	0.74 (0.40, 1.34)	0.32		
No	Reference			

Bold font indicates *p* < 0.05

## Discussion

We believe this to be the first study to examine Pap test recency and HPV vaccination among Brazilian immigrant women living in the US. The majority (83.7%) of women in the study reported having a Pap test within the past 3 years, a level similar to that in Brazil (81%) [11] and exceeding the percentage of Hispanic women (67.9%) in the U.S. classified as being up-to-date with cervical cancer screening [22]. Analysis of nationally representative data identified differences in being current with testing between women born in the U.S. (82.8%) and length of U.S. residency (66.8% for < 10 years, 77.0% ≥ 10 years) [31].

It is not surprising that women in the current study who had seen a healthcare provider in the last year were more likely to have had a Pap test within the past three years than women who had not. This finding is consistent with prior research [30], although we did not identify research specific to Brazilian immigrants in the U.S. Although most respondents reported having had a Pap test within the past three years, there is likely room for improvement. Notable barriers among immigrants to breast and cervical cancer in the U.S. include cultural and language barriers, limited education, fear, lack of transportation and time, insurance status, and financial barriers, and addressing these barriers can be beneficial [1]. The World Health Organization recommends using HPV self-sampling procedures as part of cervical cancer screening to increase screening uptake [33]. A recent meta-analysis found a positive potential effect of self-sampling among both “under-screened women (RR: 2.1; 95% CI: 1.9–2.3) and the general population (RR: 1.4; 95% CI: 1.2–1.7” and that self-sampling procedures were found to be acceptable whether conducted in a clinical and home setting [14].

Less than a third of respondents in the current study reported having received one or more doses of the HPV vaccine (30.9%). This is slightly lower than found in other studies. A 2018 study determined that 36.1% of Hispanic women had received one or more doses of the HPV vaccine [6], while a study of Hispanic adults in Indiana found that 35.6% of the sample (men and women) had received at least one dose of the HPV vaccine [28]. However, our finding that more than two-thirds of older respondents (27–46 years old) reported receiving one or more vaccine doses (64.1%) exceeds that found in other studies. For example, an analysis of nationally representative data collected in the U.S. in 2019 found that among men and women 27–45 years of age, 11.9% of Hispanic respondents had received one or more doses of the HPV vaccine compared to 15.5% of non-Hispanic Whites and 19.4% of non-Hispanic Black respondents [26]. Limited research has examined HPV vaccination among adult immigrants living in the U.S. A study among women aged 18 and 36 years found that a lower percentage of foreign-born women reported having one or more doses of the HPV vaccine compared with those that were U.S.-born (14.4% vs. 30.1%) [9]. Moreover, that study found that only 10.46% of Hispanic respondents from Mexico, Central America, and the Caribbean Islands had initiated HPV vaccination [9]. Brazilian women who emigrate to the U.S. may have—benefitted from Brazil’s nationwide efforts to increase cervical cancer screening rates in the early 2000s. Study results also suggest that there may be room to increase Pap testing in this population. However as HPV vaccination is not routinely recommended for this age group, it is not possible to conclude that more efforts are needed to promote conclude that more efforts are needed to promote shared decision-making for HPV vaccination, since we did not assess risk factors or preferences, which would be required to determine if a clinical discussion were warranted.

It should be noted that about one-third (35.8%) of the sample had not seen a healthcare provider in the previous year, and 35.5% did not have a primary care provider. This finding suggests that this population may not be receiving routine healthcare.

Given that 69.7% of participants were not vaccinated, even though vaccination was not warranted based on individual risk factors and women’s preferences, future research could explore Brazilian immigrant women’s willingness to vaccinate their children against HPV. As noted previously, the one prior study we found that focused on Brazilian immigrants in the U.S. found that most parents of teens were aware of HPV (93.6%), yet only 74.5% were aware of the HPV vaccine [18]. Therefore, efforts to educate parents about the benefits of HPV vaccination for their children may be warranted.

Study findings should be considered in the context of the limitations. First, we did not have a viable sampling frame to select Brazilian immigrant women to participate, so we used a convenience sample. Therefore, caution should be used when generalizing these findings. In addition, participants were almost entirely from Massachusetts, a progressive state where most residents are insured, and health insurance status is associated with increased healthcare access [15], as well as reduced morbidity and mortality [13]. However, racial/ethnic disparities in healthcare remain pronounced in the state [2]. Second, like other studies in this field, Pap testing and HPV vaccination status were assessed through self-report, which is less accurate than information gathered via medical records or vaccine registries [27]. Self-reported data may overestimate the recency of Pap due to recall bias and social desirability [29]. Nevertheless, self-reports are the most frequently utilized method for cancer prevention and population control research. Another limitation of this study is that we could not incorporate HPV testing to determine whether respondents were “up to date” with screening given current recommendations, which include extending Pap test intervals if done in combination with HPV testing [12] . Furthermore, we did not assess reasons for not being screened or getting the HPV vaccine. In addition, some respondents in our study may not have had the option to be vaccinated if they were age 20 or older and still living in Brazil. Despite these limitations, this study makes a unique contribution to the literature. To our knowledge, this is the first study to examine cervical cancer screening and HPV vaccination among Brazilian immigrants in the US. This is important as the Brazilian immigrant population in the US is increasing and likely differs from other Hispanic/Latino immigrant sub-populations.

## Conclusion

Our findings suggest that Brazilian women who emigrate to the U.S. may have benefitted from the strong cancer screening program in Brazil, which implemented wide-scale cervical cancer screening with Pap smears in the early 2000s [11]. Study findings suggest that there is room for improvement in terms of Pap testing. However, it is not possible to say if there is a need to do more to promote HPV vaccination in this population. Still, this study contributes new information about an understudied and growing population in the US.

## Data Availability

The datasets used and/or analyzed for the current study are available from the corresponding author upon reasonable request.

## Abbreviations

BRFSS: Behavioral Risk Factor Surveillance System
HPV: Human papillomavirus
U.S.: United States
